# Acaricidal activity of the aqueous and hydroethanolic extracts of 15 South African plants against *Rhipicephalus turanicus* and their toxicity on human liver and kidney cells

**DOI:** 10.4102/ojvr.v86i1.1665

**Published:** 2019-07-22

**Authors:** Gerda Fouche, Olubukola T. Adenubi, Tlabo Leboho, Lyndy J. McGaw, Vinny Naidoo, Kevin W. Wellington, Jacobus N. Eloff

**Affiliations:** 1 Chemistry Department, Faculty of Natural and Agricultural Sciences, University of Pretoria, Pretoria, South Africa; 2 Phytomedicine Programme, Department of Paraclinical Sciences, University of Pretoria, Onderstepoort, South Africa; 3 Council for Scientific and Industrial Research (CSIR) Biosciences, Pretoria, South Africa; 4 Biomedical Research Centre, Faculty Veterinary Science, University of Pretoria, Onderstepoort, South Africa

**Keywords:** *Rhipicephalus turanicu*s, contact bioassay, acaricidal activity, Vero cells, HepG2 cells, toxicity, water, ethanol

## Abstract

Hot water and hydroethanolic (70:30) extracts were prepared from 15 plant species, which were investigated to discover eco-friendly and less expensive tick control methods as an alternative to synthetic acaricides. A contact bioassay was used to determine the acaricidal activity of these extracts against the cattle tick, *Rhipicephalus turanicus* (Acari: Ixodidae) at a concentration of 20% (200 mg/mL). The hydroethanolic extracts had better activity than the hot water extracts against *R. turanicus*. The hydroethanolic extract from *Tabernaemontana elegans* (leaves) had the best mortality (87.0%). This was followed by *Calpurnia aurea* (stems) with a mortality of 75.0%, *Schkuhria pinnata* (whole plant) with a mortality of 67.0% and *Aloe rupestris* (leaves) with a mortality of 66.6%. The toxicity of the plant extracts was also investigated and it was found that most of the hydroethanolic and hot water extracts were either safe or very safe on human Vero kidney and liver HepG2 cells. From this study, it was evident that botanicals have the potential to be developed as environmentally benign natural acaricides against *R. turanicus*.

## Introduction

*Rhipicephalus turanicus* is a tick species found in several Mediterranean countries (Mumcuoglu et al. 1993) as well as in Africa, Asia and Europe (Li et al. 2017). Ticks are vectors of various pathogens such as bacteria, viruses and protozoa (Boldbaatar et al. 2006; Kocan, Blouin & De la Fuente 2011). *R. turanicus* is known to be a vector of rickettsial diseases such as Q-fever and North Asian tick typhus that is caused by *Rickettsia sibirica* (Berdyev 1980).

This tick species parasitises a wide range of hosts including humans and dogs. When heavy tick infestations occur in livestock, huge economic losses result through anorexia, blood loss, decrease in productivity, depression of immune function, damage to hides, general stress and irritation, toxicosis, transmission of pathogens, treatment costs and also death (Elango & Rahuman 2011; Ghosh, Azhahianambi & Yadav 2007; Tian et al. 2011). The global annual economic loss due to ticks and tick-borne diseases equates to around $7 billion (Bagavan et al. 2009; Zahir et al. 2010).

Current tick control strategies aim to reduce tick numbers to acceptable levels, reduce chemical residue risks, prevent production loss and reduce the dependence on chemical acaricides by exploiting different control treatments for different herd groups (Ghosh et al. 2007). Tick control has been accomplished by the use of chemical acaricides, which are effective when properly applied. The use of chemical acaricides has, however, resulted in environmental contamination, contamination of milk and meat products with insecticide residues and the development of acaricide-resistant ticks (Graf et al. 2004). The high cost of developing new chemical acaricides and the lack of suitable immunising agents have led to a renewed interest in the use of botanicals as an alternative for the control of cattle ticks (Madzimure et al. 2011; Zaman et al. 2012).

Botanicals have been perceived to be reasonably safe with fewer risks to the environment and also marginal impact on both human and animal health (Isman 2008; Panella et al. 2005; Su & Mulla 1998). When compared with chemical acaricides, herbal extracts have several advantages, for example, they are biodegradable, do not accumulate and pollute the environment, they are less toxic to the environment and to non-targeted species, and are also less likely to have resistance developed against them (Liang et al. 2003). There have been several reports by researchers obtaining promising results in controlling ticks by using botanicals (Cetin et al. 2009; Coskun et al. 2008; Koc et al. 2013).

After many years of displaying promise, botanicals have acquired a foothold in several markets and are finally attaining a standing with the agrochemical industry (Isman 2015). In 2011, the Environmental Protection Agency (EPA) approved Requiem^®^, an insecticide based on a terpenoid-rich extract of *Dysphania* (= *Chenopodium*) *ambrosioides* (L.) Mosyakin and Clemants (Isman 2015). Captiva^®^, an insecticide based on an extract of *Capsicum* oleoresin and garlic oil, was approved by the EPA in 2014 (Isman 2015).

In light of the above, a large number of plant extracts from several South African plants were investigated to identify novel botanicals that could be used as an alternative to synthetic acaricides against ticks. These plant species were based on a comprehensive literature survey and ethno-veterinary use by traditional communities to control ticks in livestock. In previous work, we reported on the acaricidal activity of organic extracts of South African plants against the larvae of both *Rhipicephalus decoloratus* (Koch 1844) (Acari:Ixodidae) (Fouche et al. 2016a) and *Rhipicephalus* (*Boophilus*) *microplus* (Wellington et al. 2016), and also on anthelmintic activity against *Haemonchus contortus* (Fouche et al. 2016b). Furthermore, we also reported on the acaricidal activity against *Rhipicephalus turanicus* (Fouche et al. 2017). Herein we report on the results of screening 36 extracts that were investigated for acaricidal activity against the tick species, *R. turanicus* and on their toxicity on human liver and kidney cells.

## Materials and methods

### Plant material collection

Fifteen plants (*Aloe rupestris* Baker, *Calpurnia aurea ssp. Aurea* (Aiton) Benth., *Cissus quadrangularis* L., *Cleome gynandra* L., *Clematis brachiata* Thumb., *Ficus sycomorus* L., *Monsonia angustifolia* E. Mey. ex A. Rich., *Pelargonium luridium* (Andrews) Sweet, *Schkuhria pinnata* (Lam.) Kuntze ex Thell., *Sclerocarya birrea* (A.Rich.) Hochst., *Senna italica* subsp *arachoides* (Burch.) Lock and *Tabernaemontana elegans* Stapf.) were selected on the basis of available literature and ethno-veterinary usage in South Africa.

### Production of dried, ground plant material and preparation of the hot water and hydroethanolic extracts

Plant material was dried in an oven at 30 °C – 60 °C and ground to fine particles using a hammer mill. Hot water and ethanol/water (30:70) extracts were prepared from the plants (Table 1). The hydroethanolic extract was prepared at room temperature by adding the solvent mixture (350 mL) to the ground plant material (30 g), which was followed by stirring for 1 hour. After stirring, the contents were allowed to settle and then the solvent was removed. The residue was re-extracted by adding the same volume of solvent and stirring for 1 h again. For the third extraction the same volume of solvent was used but this time the mixture was stirred overnight (24 h). After the third extraction, all the extracts were combined and the ethanol was evaporated using a rotary evaporator. A freeze drier or Genevac personal evaporator was used to remove water, and the extracts were then stored in a cold room at −20 °C.

**TABLE 1:** Plant species, plant part used for the solvent extraction, plant family, the mass and percentage of extract obtained.

Entry	Plant and plant part used in extraction	Plant family	Solvent	Extract	
				Mass (g)	%
1	*Aloe rupestris* (leaves)	Asphodelaceae	Hot water	1.0958	4
			Ethanol/water	1.1449	4
2	*Calpurnia aurea* (leaves, flowers)	Fabaceae	Hot water	1.0793	4
			Ethanol/water	1.0100	3
	*Calpurnia aurea* (stems)	Fabaceae	Hot water	1.0311	3
			Ethanol/water	1.0100	3
3	*Senna italica* subsp *arachoides* (roots, leaves, fruit)	Leguminosae	Hot water	1.1440	4
			Ethanol/water	1.1365	4
4	*Cissus quadrangularis* (stems)	Vitaceae	Hot water	1.0830	4
			Ethanol/water	1.0180	3
5	*Clematis brachiata* (whole plant)	Ranunculaceae	Hot water	1.0143	3
			Ethanol/water	1.0313	3
6	*Cleome gynandra* (leaves)	Capparidaceae	Hot water	1.0031	3
			Ethanol/water	1.0821	4
7	*Ficus sycomorus* (bark & stems)	Moraceae	Hot water	1.0322	3
			Ethanol/water	1.0186	3
8	*Gnidia deserticola* (whole plant)	Thymelaeaceae	Hot water	1.0055	3
			Ethanol/water	1.1857	4
9	*Hypoxis rigidula* (bulb)	Hypoxidaceae	Hot water	1.0507	4
			Ethanol/water	1.0439	3
10	*Maerua angiolensis* (leaves)	Capparidaceae	Hot water	1.0764	4
			Ethanol/water	1.0872	4
11	*Monsonia angustifolia* (whole plant)	Geraniaceae	Hot water	1.0516	4
			Ethanol/water	1.0709	4
12	*Pelargonium luridum* (whole plant)	Geraniaceae	Hot water	1.0252	3
			Ethanol/water	1.0212	3
13	*Schkuhria pinnata* (whole plant)	Asteraceae	Hot water	1.1300	4
			Ethanol water	1.0412	4
14	*Sclerocarya birrea* (bark, root)	Anacardiaceae	Hot water	1.0417	4
			Ethanol/water	0.9878	3
15	*Tabernaemontana elegans* (leaves)	Apocynaceae	Hot water	1.1407	4
			Ethanol/water	1.0436	4

Hot water extracts were prepared by combining ground plant material (30 g) and hot water (350 mL). The mixture was stirred while boiling at 100 °C on a hot plate for 1 h and filtered with the aid of a hydraulic pump. The concentrated extract was lyophilised to remove traces of the solvent.

### Ticks

Adult stages (both sexes) of *R. turanicus* (Acari: Ixodidae) were obtained from Clinvet International, Bloemfontein, South Africa and kept at the Phytomedicine Laboratory, Paraclinical Sciences, Faculty of Veterinary Sciences, University of Pretoria. The ticks were housed in glass humidity chambers, closed by a removable cover at an average temperature of 25 °C ± 1 °C and relative humidity of 75% ± 10%, which was maintained by placing saturated sodium chloride solution in the glass chamber. The vials, in which the ticks were stored, were covered with cotton mesh (to allow normal air exchange) and set on a square glass plate placed at the base of the chamber on four small bearings so that the edges of the plate were at a distance of 1.5 cm from the walls. The saturated saline solution on the floor of the glass chamber also prevented the ticks from reaching the walls (Zorloni, Penzhorn & Eloff 2010).

### Determination of the acaricidal activity

The contact bioassay described by Zorloni et al. (2010) was employed. One microliter of a 20% (200 mg/mL) concentration of each extract was dropped on the dorsum of each tick (*n* = 10) for 1 minute before storing them in a vial covered with a perforated stopper. The same procedure was followed for the negative control (acetone or distilled water) and positive control (cypermethrin, 5 mg/mL). Each treatment was performed in triplicate on each of three different occasions to yield nine replicates. The percentage mortality was determined 24 h after exposure by observing under a stereomicroscope (American Optical Corporation). The ticks were recorded as alive and active if they exhibited normal behaviour on exposure to carbon dioxide (CO_2_) from human breath (host-associated stimulus) or after being physically stimulated with plastic tweezers, whereas they were confirmed dead based on signs of cuticle darkness, halted Malpighian tubules movement and haemorrhagic skin lesions. Those showing some difficulty in movement or maintaining a normal posture were termed weak or very weak if there was no leg coordination or a loss of a righting reflex (a reflex that corrects the orientation of the body when it is taken out of its normal upright position). The percentage mortality in all the experimental groups was corrected by applying Abbott’s formula (Abbott 1987):

$$\text{Corrected percent mortality }\%=\frac{\%\text{ test mortality}-\%\text{ control mortality}}{100-\%\text{ control mortality}}\times 100$$ [Eqn 1]

### Determination of the toxicity of the plant extracts

Viable cell growth after incubation of Vero African Green monkey kidney cells (ATCC^®^ CCL-81^TM^) and HepG2 human liver cancer cells (ATCC^®^ HB-8065^TM^) with test samples was determined using the tetrazolium-based (MTT) colorimetric assay to determine the cell viability (McGaw, Steenkamp & Eloff 2007; Mosmann 1983). For Vero cells, minimum essential medium (MEM) was used as a growth medium and HepG2 cells were cultured in Dulbecco’s minimum essential medium (DMEM). Doxorubicin hydrochloride (Pfizer) was used as a positive control. Each plant extract was tested in quadruplicate and the assays were repeated three times (McGaw et al. 2007; Mosmann 1983).

### Statistical analysis

Data on tick mortality and cytotoxicity were presented as the arithmetic mean values ± standard error of the mean (Mean ± SEM). Significance was analysed using one-way analysis of variance followed by Tukey’s multiple comparison test on GraphPad Prism 7.02 (GraphPad Software, San Diego, CA). Values were considered to differ statistically when *p* ≤ 0.05.

### Ethical considerations

This article followed all ethical standards for research without direct contact with human or animal subjects.

## Results

### Extraction and yield

The assayed plants represent 13 plant families with the Capparidaceae and Geraniaceae families being represented by two plant species. The leaves were the most common plant part used followed by the whole plant, stem, bark, root and fruit (Table 1). The mass that was obtained for each of the extracts is shown in Table 1. From 30 g ground plant material 3% – 4% yields were obtained.

### Determination of the acaricidal activity

The method used to determine the acaricidal activity of the plant extracts was the contact bioassay described by Zorloni (2008). The plant extracts were exposed *in vitro* to the adults of *R. turanicus* for efficacy testing. The pyrethroid insecticide, cypermethrin, was used as a positive control.

The hydroethanolic extracts had higher activity than the hot water extracts. *T. elegans* (leaves) had the best mortality (87.0%). This was followed by *C. aurea* (stems) with a mortality of 75.0%, *S. pinnata* (whole plant) with a mortality of 67% and *A. rupestris* (leaves) with a mortality of 66.6%, as shown in Table 2.

**TABLE 2:** The mortality, corrected mortality and toxicity of the fifteen indigenous South African plant species screened against *R. turanicus*.

Entry	Plant and plant part	Solvent	Mortality	CM (%)	Vero cells LC_50_ (µg/mL)	HepG2 LC_50_ (µg/mL)
1	*Aloe rupestris* (leaves)	Hot water	N.D.	N.D.	107 ± 1§§	459 ± 12§§
		Ethanol/water	70.0‡	66.6¶	153 ± 5§§	496 ± 11§§
2	*Calpurnia aurea* (stems)	Hot water	45.0§	38.9	N.D.	N.D.
		Ethanol/water	77.5‡	75.0‡	N.D.	N.D.
	*Calpurnia aurea* (leaves, flowers)	Hot water	64.0¶	60.0¶	> 1000‡‡	> 1000‡‡
		Ethanol/water	25.0††	16.7††	> 1000‡‡	> 1000‡‡
3	*Senna italica* subsp *arachoides* (roots, leaves, fruit)	Hot water	35.0††	27.8††	> 1000‡‡	> 1000‡‡
		Ethanol/water	27.5††	19.4††	> 1000‡‡	601 ± 36¶¶
4	*Cissus quadrangularis* (stems)	Hot water	30.0††	22.2††	> 1000‡‡	> 1000‡‡
		Ethanol/water	22.5††	13.8††	806 ± 1¶¶	618 ± 16*¶¶
5	*Cleome gynandra* (leaves)	Hot water	35.0††	27.8††	> 1000‡‡	> 1000‡‡
		Ethanol/water	37.5††	30.5††	> 1000‡‡	> 1000‡‡
6	*Clematis brachiata* (whole plant)	Hot water	37.5††	30.5††	> 1000‡‡	568 ± 35¶¶
		Ethanol/water	40.0††	33.5††	857 ± 11¶¶	683 ± 17¶¶
7	*Ficus sycomorus* (bark & stems)	Hot water	22.5††	13.8††	> 1000‡‡	> 1000‡‡
		Ethanol/water	25.0††	16.7††	641 ± 5¶¶	708 ± 42¶¶
8	*Gnidia deserticola* (whole plant)	Hot water	57.5§	53.0§	> 1000‡‡	> 1000‡‡
		Ethanol/water	37.5††	31.0††	946 ± 2¶¶	233 ± 1§§
9	*Hypoxis rigidula* (bulb)	Hot water	N.D	N.D.	> 1000§	> 1000§
		Ethanol/water	7.5††	0.0††	> 1000‡‡	740 ± 29*¶¶
10	*Maerua angiolensis* (leaves)	Hot water	N.D	N.D.	> 1000‡‡	> 1000‡‡
		Ethanol/water	25.0††	16.7††	27 ± 6*†††	> 1000‡‡
11	*Monsonia angustifolia* (whole plant)	Hot water	25.0††	16.7††	> 1000‡‡	> 1000‡‡
		Ethanol/water	27.5††	19.4††	241 ± 50§§	3*†††
12	*Pelargonium luridum* (whole plant)	Hot water	27.5††	19.4††	569 ± 6¶¶	369 ± 21* §§
		Ethanol/water	2.5††	0.0††	595 ± 4¶¶	330 ± 24*§§
13	*Schkuhria pinnata* (whole plant)	Hot water	**57.5§**	**53.0§**	655 ± 32¶¶	532 ± 40¶¶
		Ethanol/water	**71.0†**	**67.0¶**	460 ± 7§§	116 ± 40*§§
14	*Sclerocarya birrea* (bark, root)	Hot water	7.5††	0.0††	**> 1000**‡‡	**> 1000**‡‡
		Ethanol/water	22.5††	13.8	**>1000**‡‡	**> 1000**‡‡
15	*Tabernaemontana elegans* (leaves)	Hot water	N.D.	N.D.	69 ± 2*†††	418 ± 4§§
		Ethanol/water	**88.2†**	**87.0†**	49 ± 5*†††	**71 ± 7**†††
16	Positive control (cypermethrin)	-	**100.0†**	-	-	-
17	Negative control (acetone only)	-	10.0††	-	-	-
18	Negative control (distilled water only)	-	0.0††	-	-	-
19	Positive control (doxorubicin)	-	-	-	**1.52 ± 0.3**†††	0.34 ± 0.14†††

Note: GraphPad Instat 3.0 Student-Newman-Kuels was used to analyse the difference of the same extract on two different cell lines.

N.D., not determined; CM, corrected mortality.

* , Statistically different (*p* < 0.05).

Acaricidal activity:

† , Very good (> 80%);

‡ , Good (70%–80%);

¶ , moderate: (60% – 69%);

§ , poor (40% – 59%);

†† , Very poor (< 40%).

Toxicity:

‡‡ , very safe (LC_50_ > 1000 *µ*g/mL);

¶¶ , safe (LC_50_ = 500–1000 *µ*g/mL);

§§ , slightly toxic (LC_50_ = 100–500 *µ*g/mL);

††† , toxic (LC_50_ < 100 *µ*g/mL).

The extract of *C. aurea* (leaves, flowers) had the best acaricidal activity (60%), which was followed by *S. pinnata* (whole plant) and *G. deserticola* (whole plant) each with an activity of 53%. Poor activity (10% – 47%) was observed for the remainder of the hot water extracts as shown in Table 2.

### Determination of the toxicity of the plant extracts on Vero cells

A cut-off of 100 *µ*g/mL was used for toxicity (Nondo et al. 2015) and, therefore, many of the extracts of the plants had low toxicity on Vero cells as the LC_50_ values were greater than 100 *µ*g/mL. The LC_50_ values were compared with that of the positive control, doxorubicin (LC_50_ = 1.52 *µ*g/mL), as shown in Table 2. The hydroethanolic (LC_50_ = 49 *µ*g/mL) and hot water (LC_50_ = 69 *µ*g/mL) extracts of *T. elegans* were toxic to Vero cells.

### Toxicity on HepG2 cells

Only the hydroethanolic extract of *M. angustifolia* and *T. elegans* with LC_50_ values of 3.0 *µ*g/mL and 71.0 *µ*g/mL compared with the positive control, doxorubicin (LC_50_ = 0.34 *µ*g/mL), which was toxic as shown in Table 2. Overall, for most of the plants, the LC_50_ values were greater than 1000 *µ*g/mL and were therefore classified to be safe.

### Comparative toxicity of hydroethanolic and hot water extracts on Vero and HepG2 cells

It was apparent that for many of the plants the hydroethanolic and hot water extracts were more toxic to the HepG2 cells than the Vero cells (Table 2). For *H. rigidula, M. angustifolia, P. luridum, S. pinnata* and *S. italica*, there was a significant statistical difference.

## Discussion

It is evident from the results in Table 2 that the hydroethanolic extracts had better mortality against ticks than the hot water extracts. Furthermore, most of the hydroethanolic and hot water extracts were either safe or very safe on Vero and HepG2 cells.

The best mortality (87%) was observed for the hydroethanolic extract of *T. elegans* (leaves), which was also toxic (LC_50_ < 100 *µ*g/mL) to Vero (LC_50_ = 49 ± 5 *µ*g/mL**)** and HepG2 (LC_50_ = 71 ± 7 *µ*g/mL) cells. Phytochemical research has shown that *T. elegans* is rich in monoterpenoid indole alkaloids such as coronaridine, voacangine, hydroxycoronaridine, isovoacangine, 11-hydroxycoronaridine, voacristine, 19-epi-voacristine, isovoacristine, ibogamine, 10-methoxyibogamine, 11-methoxyibogamine and 19-epi-heyneanine (Bombardelli et al. 1976; Danieli et al. 1980; Gabetta, Martinelli & Mustich 1975; Kam & Sim 2002; Prakash Chaturvedula et al. 2003; Van der Heijden, Brouwer & Verpoorte 1986). A range of biological activities have been reported for these indole alkaloids such as anticholinesterase, antihypertensive, antimicrobial and antimalarial properties, central nervous system-stimulating activity and particularly antitumor activity (Andrade et al. 2005; De Almeida et al. 2004; Delorenzi et al. 2001; Kam et al. 2004; Medeiros et al. 2011; Perera et al. 1985; Prakash Chaturvedula et al. 2003; Van Beek et al. 1984; Vieira et al. 2008). *β*-Carboline indole alkaloids have been isolated from the methanol leaf extract of *T. elegans* (Mansoor et al. 2009). It is likely that alkaloids may also have been extracted by the hydroethanolic extract which may, therefore, be responsible for the acaricidal activity against *R. turanicus*.

Acaricidal activity was also noted for the hydroethanolic extract of *Calpurnia aurea* (stems) and the hot water extract of *C. aurea* (leaves, flowers), which had mortality values of 75% and 60%, respectively. The latter was found to be very safe (LC_50_ > 100 µg/mL) against both Vero and HepG2 cells. Our results were in agreement with those of Zorloni et al. (2010) who also reported acaricidal activity for this plant species but for acetone leaf extracts. In their report, 20% and 10% acetone leaf extracts of *C. aurea* either killed or severely compromised the mobility of unfed adults of *Rhipicephalus pulchellus* ticks.

The main chemical constituents of *C. aurea* are phenolic compounds (Adedapo et al. 2008), which are said to be accountable for the attraction behaviour of over 12 species of ixodid ticks (McDowell & Wallade 1986; Wood et al. 1975; Yoder & Stevens 2000). The efficacy of the extract of *C. aurea* (leaves) may be ascribed to its capacity to lure and also both kill or ruthlessly compromise the mobility of ticks.

Zorloni (2008) reported that *C. aurea* used to control ticks on animals in southern Ethiopia had comparable acaricidal activities to the plant in South Africa despite growing under widely diverse environmental conditions.

The hydroethanolic extract of *Aloe rupestris* (leaves) had the second best mortality (66.6%) but was found to be slightly toxic (LC_50_ = 100–500 *µ*g/mL) against Vero (LC_50_ = 153 ± 5 *µ*g/mL) and HepG2 cells (LC_50_ = 496 ± 11 *µ*g/mL). This acaricidal activity correlates with a report on that of another *Aloe* species, *Aloe ferox*, which has been used as a tick control remedy in some South African villages (Moyo & Masika 2009). *Aloe* species have also been used in Ethiopia to control ectoparasites (Gemeda et al. 2014).

It has been shown by phytochemical analyses that numerous *Aloe* species contain diverse carbohydrate polymers (notably glucomannans) and an array of other low molecular weight phenolic compounds comprising alkaloids, anthraquinones, anthrones, benzene and furan derivatives, chromones, coumarins, flavonoids, phytosterols, pyrans and pyrones (Cock 2015). 7-*O*-Methylaloesin was isolated from the leaf exudate of *A. rupestris* (Bisrat et al. 2000).

The hydroethanolic extract of *S. pinnata* (whole plant) had a mortality of 67% against *R. turanicus* and was found to be slightly toxic (LC_50_ = 100–500 *µ*g/mL) against both Vero (LC_50_ = 460 ± 7 *µ*g/mL) and HepG2 cells (LC_50_ = 116 ± 40 *µ*g/mL). This plant and its varieties have been used as either insect repellents or insecticides mostly against fleas (Heiser 1945). Phytochemical investigations have afforded polyacetylenes (Bohlmann & Zdero 1977), diterpenes (Bohlmann et al. 1980), sesquiterpene lactones (Delgado, Hernández & Romo De Vivar 1984; Herz & Govindan 1980; Pérez et al. 1984; Pettei et al. 1978) and phenylpropanoids (Delgado & Tejeda 1998). Some of these compounds have biological activities (Delgado et al. 1998). These compounds may, therefore, be responsible for the observed acaricidal activity against *R. turanicus*.

Both the hot water and hydroethanolic extract of *G. deserticola* (whole plant) had poor activity of 53% and 16.7%, respectively, against *R. turanicus*. The hot water extract was found to be very safe (LC_50_ > 1000 *µ*g/mL) against both Vero and HepG2 cells. It has been found in phytochemical investigations that the genus *Gnidia* is abundant in chromones, coumarins, diterpene esters, flavonoids, lignans and neolignans (Bhandurge et al. 2013).

None of the extracts of *Senna italica* subsp *arachoides* (roots, leaves, fruits) had efficacy against *R. turanicus*. Acaricidal activity was reported for the root extract of *S. italica* subsp. a*rachoides* against adults of *Hyalomma rufipes* with the ethyl acetate extract being the most potent out of several extracts (hexane, chloroform, dichloromethane, ethyl acetate and methanol) tested (Magano et al. 2008).

For *C. quadrangularis* (stem), the hot water and hydroethanolic extracts had poor activity (22.2% and 13.8%, respectively) against *R. turanicus.* This is in contrast to what was reported by Santhoshkumar et al. (2012) who found that the aqueous extract of *C. quadrangularis* (stem) had acaricidal activity against *R. (B.) microplus*. Furthermore, the aqueous stem extract attached to silver nanoparticles were found to be more active against *R. (B.) microplus* than the free aqueous stem extract.

Similarly, the hot water and hydroethanolic extracts of *C. gynandra* (leaves) had poor activity (27.8% and 30.5%, respectively). Malonza et al. (1992), however, reported on the acaricidal activity of the leaf extract of this plant but against nymphal and adult *Amblyomma variegatum* and *R. appendiculatus* tick species.

From the results of screening the extracts against Vero and HepG2 cells (Table 2) it is evident that none were as toxic as the positive control, doxorubicin (Vero cells: LC_50_ = 1.52 *µ*g/mL ± 0.32 *µ*g/mL; HepG2: LC_50_ = 0.34 *µ*g/mL ± 0.14 *µ*g/mL).

The use of ethno-veterinary leads for investigating plants for acaricidal activity against ticks has compounded the probability of discovering novel, natural acaricides capable of combatting *R. turanius* and also other tick species. Botanicals with validated bioactivity and little to no toxicity, may have commercial value to the livestock industry.

## Conclusion

The hydroethanolic extracts of *T. elegans* (leaves), *A. rupestris* (leaves), *C. aurea* (stems) and *S. pinnata* have good to very good acaricidal activity against *R. turanicus* but are slightly toxic or toxic to Vero and HepG2 cells. The hot water extracts of *C. aurea* (leaves and flowers), *G. deserticola* (whole plant), and *S. pinnata* (whole plant) have poor acaricidal activity (53% – 60%) but are safe or very safe on Vero and HepG2 cells.

From these results, it is evident that these plant extracts have potential as acaricidal agents against *R. turanicus.* Compound isolation from these plant extracts could result in the discovery of novel natural acaricidal agents less toxic to non-targeted species and the environment.
