# Corrigendum: Acaricidal activity of the aqueous and hydroethanolic extracts of 15 South African plants against *Rhipicephalus turanicus* and their toxicity on human liver and kidney cells

**DOI:** 10.4102/ojvr.v88i1.1951

**Published:** 2021-07-27

**Authors:** Gerda Fouche, Olubukola T. Adenubi, Tlabo Leboho, Mbokota C. Khosa, Lyndy J. McGaw, Vinny Naidoo, Kevin W. Wellington, Jacobus N. Eloff

**Affiliations:** 1 Chemistry Department, Faculty of Natural and Agricultural Sciences, University of Pretoria, Pretoria, South Africa; 2 Phytomedicine Programme, Department of Paraclinical Sciences, University of Pretoria, Onderstepoort, South Africa; 3 Council for Scientific and Industrial Research (CSIR) Biosciences, Pretoria, South Africa; 4 Agricultural Research Council – Tropical and Subtropical Crops, Nelspruit, South Africa; 5 Biomedical Research Centre, Faculty Veterinary Science, University of Pretoria, Onderstepoort, South Africa

In the published version of this article, Fouche, G., Adenubi, O.T., Leboho, T., McGaw, L.J., Naidoo, V., Wellington, K.W. et al., 2019, ‘Acaricidal activity of the aqueous and hydroethanolic extracts of 15 South African plants against *Rhipicephalus turanicus* and their toxicity on human liver and kidney cells’, *Onderstepoort Journal of Veterinary Research* 86(1), a1665. https://doi.org/10.4102/ojvr.v86i1.1665, the fourth author, Mbokota C. Khosa, was omitted from the ‘Authors’ and ‘Affiliations’ sections. The indicated author should be added as the fourth author, and the following affiliation should be added as his affiliation: Agricultural Research Council – Tropical and Subtropical Crops, Nelspruit, South Africa.

The Authors’ contributions section is hereby update to:

## Authors’ contributions

G.F. conceptualised the study. M.C.K. was involved in the collection of some of the plant material and in the preparation of the extracts used in the biological screening assays. G.F., K.W.W. and T.L. performed the literature search and plant selection. T.L. prepared the plant extracts. M.C.K. was also involved in the fractionation and isolation process in the natural product chemistry laboratory. J.N.E. conceptualised the study in a joint application, and supervised the students and postdoctoral fellow. V.N. supervised determination of acaricidal activity. L.J.M.G. supervised the determination of cytotoxicity. O.T.A. determined the acaricidal activity against adult ticks of *R. turanicus*. K.W.W. wrote the first draft of the manuscript.

